# First-in-human administration of [^161^Tb]Tb-SibuDAB and comparative dosimetry with standard [^177^Lu]Lu-PSMA-I&T as part of the PROGNOSTICS phase Ia study

**DOI:** 10.1007/s00259-024-07009-w

**Published:** 2024-12-05

**Authors:** Alin Chirindel, Guillaume P. Nicolas, Frida Westerbergh, Lisa McDougall, David E. Schmid, Susanne Geistlich, Viviane J. Tschan, Sarah D. Busslinger, Angelique Fokkema, Nicola Aceto, Peter Bernhardt, Nicholas P. van der Meulen, Cristina Müller, Damian Wild, Roger Schibli

**Affiliations:** 1 Division of Nuclear Medicine, University Hospital Basel, Petersgraben 4, Basel, CH-4031 Switzerland; 2 Department of Medical Radiation Sciences, Institute of Clinical Sciences, Sahlgrenska Academy at University of Gothenburg, Gothenburg, Sweden; 3 PSI Center for Life Sciences, Villigen-PSI, Canton of Aargau, Switzerland; 4 Department of Biology, Institute for Molecular Health Sciences, ETH Zurich, Zurich, Switzerland; 5 Department of Medical Physics and Biomedical Engineering, Sahlgrenska University Hospital, Gothenburg, Sweden; 6 PSI Center for Nuclear Engineering and Sciences, Villigen-PSI, Canton of Aargau, Switzerland; 7 Department of Chemistry and Applied Biosciences, ETH Zurich, Zurich, Switzerland

We present results from the first patient in the PROGNOSTICS (NCT06343038) Phase Ia study, a prospective, single-center, single-blind trial aiming to translate [¹⁶¹Tb]Tb-SibuDAB to clinical radioligand therapy (RLT).

While ¹⁷⁷Lu-based RLT effectively treats metastatic castration-resistant prostate cancer (mCRPC), responses are often inconsistent and short-lived. Disease relapse and progression may be attributed to micrometastases which are not adequately irradiated due to the decay characteristics of ^177^Lu [1].

Preclinical studies show that ^161^Tb has superior tumor cell-killing effects compared to ^177^Lu, offering additional therapeutic benefits from short-range conversion and Auger electrons [2]. The novel radioligand [¹⁶¹Tb]Tb-SibuDAB, designed with albumin-binding properties for prolonged circulation, enhances tumor accumulation and radiation, particularly on micrometastases. Current preclinical data suggest [¹⁶¹Tb]Tb-SibuDAB may improve RLT efficacy for mCRPC [3].

In Phase Ia of the PROGNOSTICS study, mCRPC patients with stable disease on [¹⁷⁷Lu]Lu-PSMA-I&T RLT receive two test injections: 1 GBq [¹⁷⁷Lu]Lu-PSMA-I&T and 1 GBq [¹⁶¹Tb]Tb-SibuDAB, administered 3 weeks apart in random order. Quantitative SPECT/CT imaging was performed at 3, 24, 48, and 168 h post-injection. Tumor and organ absorbed doses were computed using a 75 keV ± 10% window for ^161^Tb (low-energy high-resolution collimator) and a 208 keV ± 10% window for ^177^Lu (medium-energy low-penetration collimator), using a Monte Carlo OSEM reconstruction algorithm. Safety and acute toxicity (CTCAE 5.0) along with transcriptional “omics” and circulating tumor cells were evaluated after each test injection.

The first patient (85y) enrolled was diagnosed in 2014 with T3N1M1, Gleason 9, iPSA 70 ng/ml, treated with ADT and Enzalutamide, found unfit for chemotherapy therefore receiving 3 cycles of standard [^177^Lu]Lu-PSMA-I&T.

The figure below provides a head-to-head comparison after test injections in this patient (tumors marked with red arrows). Equal gray-scale windowing was applied, with voxel values expressed in kBq. Compared to [^177^Lu]Lu-PSMA-I&T, [^161^Tb]Tb-SibuDAB resulted in longer mean tumor effective half-live (T_h_ = 135 h vs. 67 h, respectively) and increased mean tumor absorbed dose (D = 6.5 vs. 2.6 Gy/GBq, respectively).

The image-based dosimetry revealed slightly higher organ absorbed doses (0.5 vs. 0.3 Gy/GBq for parotid glands and 2.6 vs. 1.2 Gy/GBq for kidneys), yet tumor-to-organ dose ratios favor [^161^Tb]Tb-SibuDAB. The blood-based bone marrow absorbed dose was 0.11 vs. 0.02 Gy/GBq, with no acute toxicity or significant adverse effect (maximal grade 2) observed for the 6-week follow-up period.

**Figure:**
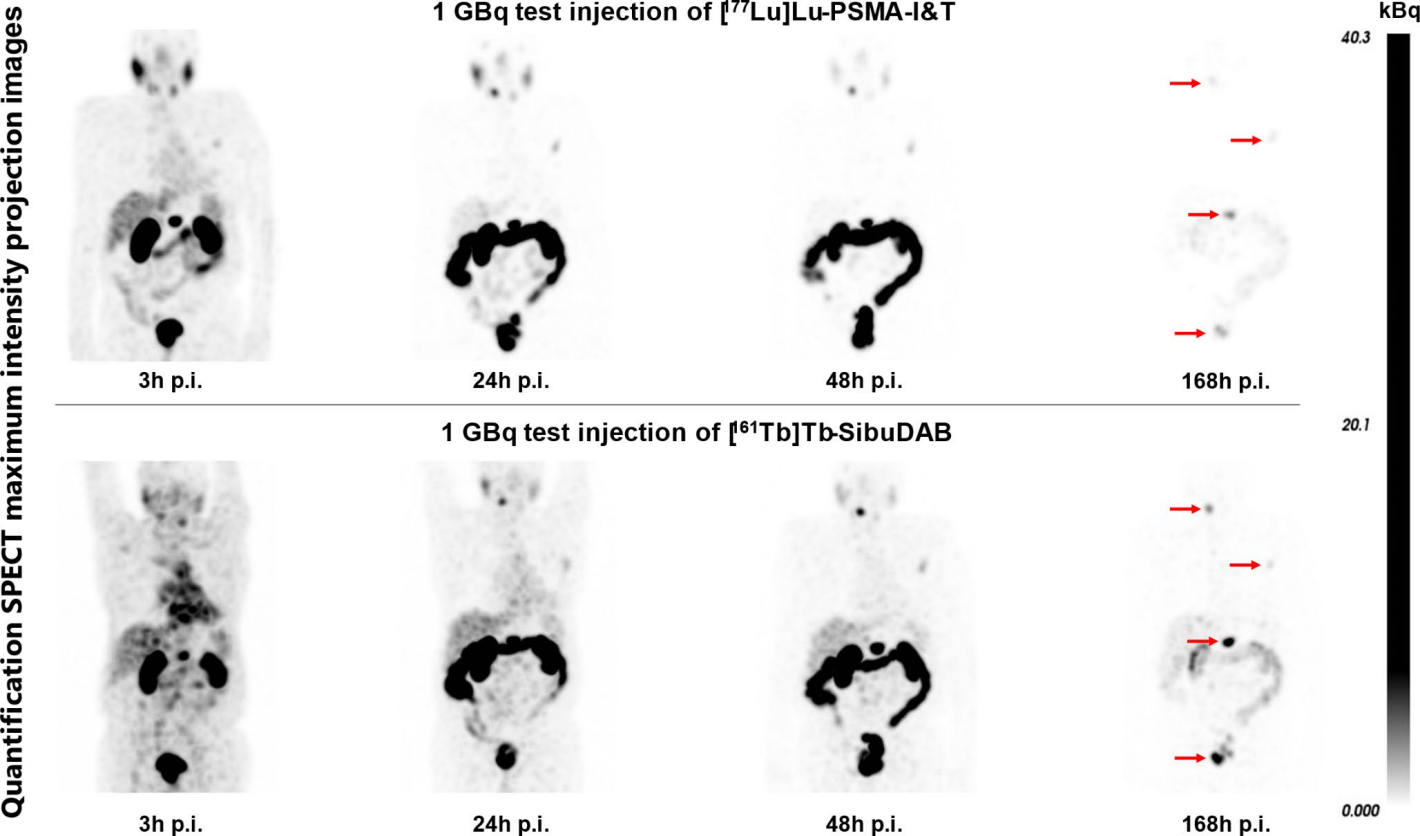

First-in-human data suggest [^161^Tb]Tb-SibuDAB is a promising radiopharmaceutical for the current indication of RLT in mCRPC. The potential to enhance treatment durability against micrometastases could be further explored in a neo/adjuvant setting. The planned dose escalation study (Phase Ib of PROGNOSTICS) will assess the efficacy, toxicity, and therapeutic index of [¹⁶¹Tb]Tb-SibuDAB

## Data Availability

Raw data will be made available upon reasonable request.
